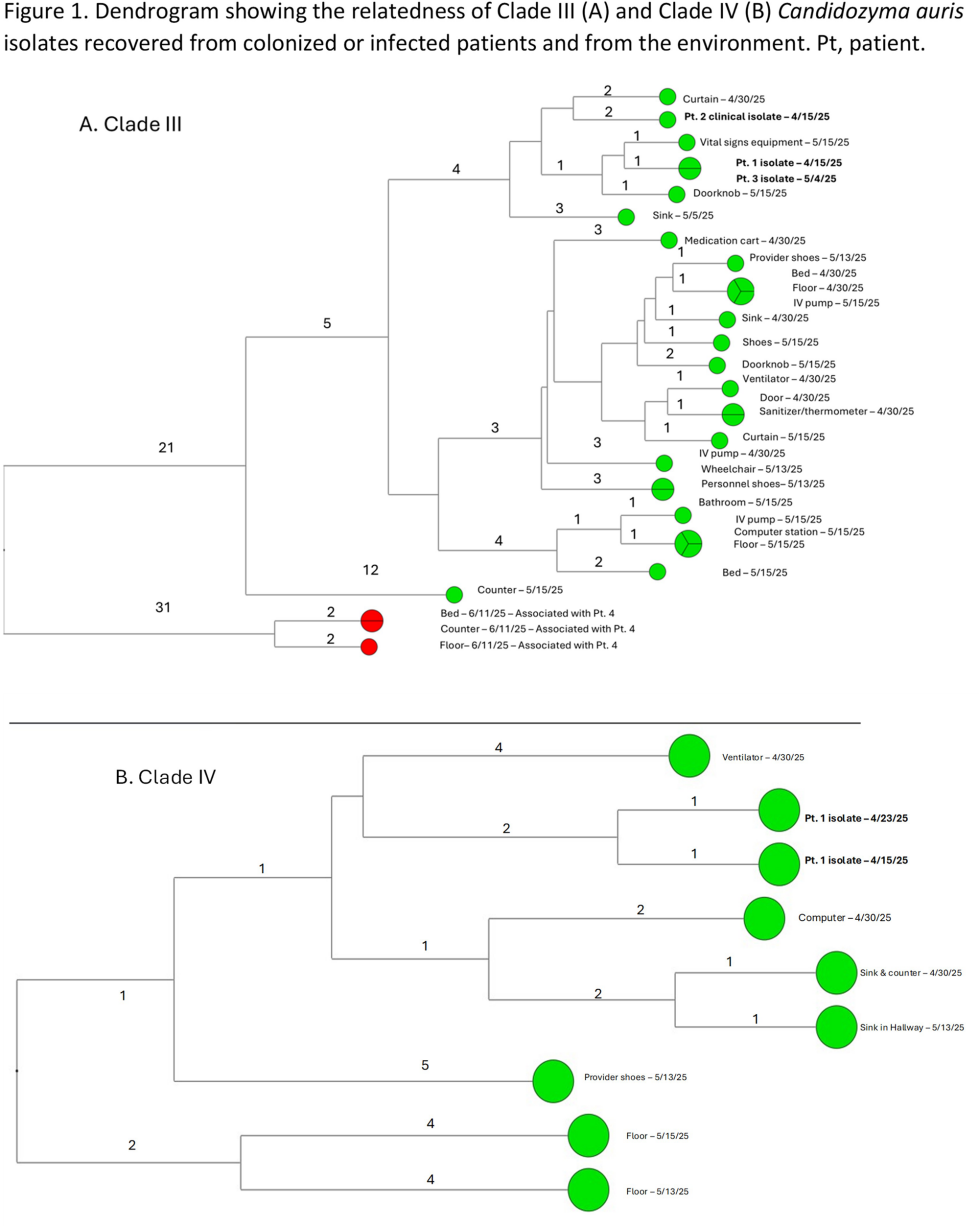# 28 Optimizing Pediatric ICU Central Line Maintenance and Removal Through Weekly Multidisciplinary CLABSI Prevention Rounds

**DOI:** 10.1017/ash.2026.10472

**Published:** 2026-06-23

**Authors:** Jose Andres Portillo, Jennifer Cadnum, Mervat Elebrashy, Munok Hwang, Hosoon Choi, Piyali Chatterjee, Chetan Jinadatha, Tina Lewis, Jay Krishnan, Curtis Donskey, Elie Saade, University Hospitals Cleveland Medical Center

**Affiliations:** 1 University Hospitals; 2 Cleveland VA Medical Center; 3 Central Texas VA Research Foundation; 4 Central Texas Veterans Health Care System; 5 University Hospitals Health System; 6 University Hospitals, Case Western Reserve University; 7 Case Western Reserve University

## Abstract

**Background:** Candidozyma auris is an emerging multidrug-resistant pathogen characterized by environmental persistence and healthcare-associated transmission. Genomic epidemiology can help clarify transmission networks linking clinical isolates, shared equipment, and contaminated surfaces. **Methods:** We integrated environmental surveillance culturing with whole-genome sequencing (WGS) to characterize environmental reservoirs and genomic diversity of C. auris at University Hospitals Cleveland Medical Center, a tertiary-care hospital. Environmental surveillance samples were collected from high-touch surfaces, shared equipment, and other environmental sites within patient care areas and C. auris clinical isolates were obtained. All C. auris isolates underwent whole-genome sequencing. Dendrograms were constructed using single nucleotide polymorphism (SNP) distances to assess genetic relatedness (isolates with <13 SNP differences were considered related) and evaluate overlap between clinical and environmental isolates to infer transmission-relevant patterns. **Results:** Two genetically distinct C. auris clades, Clade III and Clade IV, circulated concurrently in April to May 2025. Three patients were infected with genomically related Clade III isolates (Figure 1.A) and 3 were infected with related Clade IV isolates (Figure 1.B). For both clades, multiple related isolates were present in the environment, including patient room surfaces, portable equipment, floors, and shoes of personnel. For Clade III, a distinct isolate (31 SNP difference from prior isolates) was detected in the environment in June 2025 with no concurrent clinical isolates. **Conclusions:** Our findings are consistent with previous evidence that high-touch surfaces and portable equipment frequently become contaminated with C. auris and suggest that floors and shoes may be an underappreciated source of dissemination. Integrating environmental surveillance with whole-genome sequencing can help identify likely reservoirs, clarify transmission dynamics, and guide targeted infection prevention interventions.

**Figure:**